# Genetic parameters and genome-wide association study of hyperpigmentation of the visceral peritoneum in chickens

**DOI:** 10.1186/1471-2164-14-334

**Published:** 2013-05-16

**Authors:** Chenglong Luo, Hao Qu, Jie Wang, Yan Wang, Jie Ma, Chunyu Li, Chunfen Yang, Xiaoxiang Hu, Ning Li, Dingming Shu

**Affiliations:** 1 Institute of Animal Science, Guangdong Academy of Agricultural Sciences, 1 Dafeng 1st Street, Wushan, Tianhe District, Guangzhou, Guangdong, 510640, China; 2 State Key Laboratory for Agro-Biotechnology, China Agricultural University, Beijing, 100193, China; 3 State Key Laboratory of Livestock and Poultry Breeding, Guangzhou, 510640, China

## Abstract

**Background:**

Hyperpigmentation of the visceral peritoneum (HVP) has recently garnered much attention in the poultry industry because of the possible risk to the health of affected animals and the damage it causes to the appearance of commercial chicken carcasses. However, the heritable characters of HVP remain unclear. The objective of this study was to investigate the genetic parameters of HVP by genome-wide association study (GWAS) in chickens.

**Results:**

HVP was found to be influenced by genetic factors, with a heritability score of 0.33. HVP had positive genetic correlations with growth and carcass traits, such as leg muscle weight (r_g_ = 0.34), but had negative genetic correlations with immune traits, such as the antibody response to Newcastle disease virus (r_g_ = −0.42). The GWAS for HVP using 39,833 single nucleotide polymorphisms indicated the genetic factors associated with HVP displayed an additive effect rather than a dominance effect. In addition, we determined that three genomic regions, involving the 50.5–54.0 Mb region of chicken (*Gallus gallus*) chromosome 1 (GGA1), the 58.5–60.5 Mb region of GGA1, and the 10.5–12.0 Mb region of GGA20, were strongly associated (*P* < 6.28 × 10^-7^) with HVP in chickens. Variants in these regions explained >50% of additive genetic variance for HVP. This study also confirmed that expression of *BMP7*, which codes for a bone morphogenetic protein and is located in one of the candidate regions, was significantly higher in the visceral peritoneum of Huiyang Beard chickens with HVP than in that of chickens without pigmentation (*P* < 0.05).

**Conclusions:**

HVP is a quantitative trait with moderate heritability. Genomic variants resulting in HVP were identified on GGA1 and GGA20, and expression of the *BMP7* gene appears to be upregulated in HVP-affected chickens. Findings from this study should be used as a basis for further functional validation of candidate genes involved in HVP.

## Background

**Figure 1:**
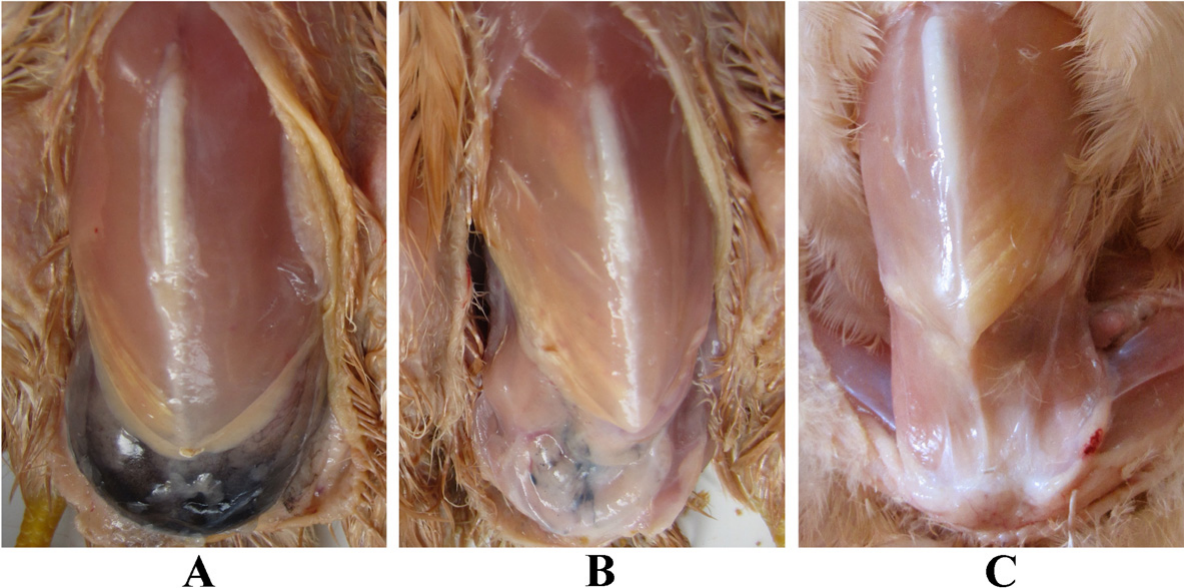
**Classification of the hyperpigmentation of visceral peritoneum. A**, **B**, and **C** represent severe, mild, and absent hyperpigmentation of the visceral peritoneum, respectively.

Pigmentation is widespread amongst both plants and animals, and plays important roles in photosynthesis (in plants), camouflage, sex selection, and protection from sunburn. However, abnormal pigmentation in humans and other animals, including hyperpigmentation (e.g. chloasma [1] and melanoma [2,3]) and the absence of pigmentation (e.g. albinism [4] and vitiligo [5,6]), can pose serious health risks. Most pigmentation phenotype variants are affected by genetic factors in both humans [7-9] and animals [10-17]. In chickens, a mutation of the melanocortin 1 receptor (*MC1R*) gene causes extended dark feathers [15], and a complex genomic rearrangement on chicken (*Gallus gallus*) chromosome 20 (GGA20) determines dermal hyperpigmentation [14]. However, these studies have mainly focused on pigmentation of the retina, skin, hair, and feathers. Pigmentation of other tissues, including muscular and visceral membranes, is also very important. Recently, more attention has been paid to the hyperpigmentation of the visceral peritoneum (HVP) in chickens, especially the colored chicken breeds, because it affects the carcass appearance of commercial chickens, resulting in economic losses, and may be associated with certain diseases, including melanomas [18]. HVP is similar to fibromelanosis, but the pigmentation is limited to the chicken peritoneum, so it may be peritoneal fibrosis. It is characterized by intense pigmentation of connective tissue in the visceral peritoneum, which results in a dark blue appearance through the skin of the chicken abdomen, and a black connective tissue layer when the skin is removed (Figure 1). A preliminary study showed that HVP was caused by an abnormal distribution of melanin, and that the number of chickens with HVP can increase in cold and humid environments (unpublished data). HVP is distinct from fibromelanosis, which results from a complex genomic rearrangement involving the endothelin 3 (*EDN3*) locus [14]. In fact, the genetic causes of HVP remain unknown.

The development of molecular tools and strategies has allowed the investigation of the genetic basis of HVP. Genome-wide association studies (GWAS) have become an important strategy for investigating the genetic basis of many human diseases, including diabetes, breast cancer, pancreatic cancer, and hypertension, amongst others (http://www.genome.gov/GWAStudies). Livestock breeders have begun to implement GWAS to map economically important quantitative trait loci (QTLs) [19-22]. Significant loci linked to chicken growth traits have been mapped to GGA1 and GGA4 by GWAS [23,24]. Therefore, if HVP is influenced by a major genetic factor, GWAS may be able to dissect its genetic basis.

In this study, we estimated the genetic parameters of HVP to illustrate the inheritance of HVP, and carried out a GWAS analysis of HVP using the chicken 60K single nucleotide polymorphism (SNP) panel in a commercial chicken population with a rich diversity of HVP.

## Results

### Genetic parameters

HVP was found to have a moderate heritability (h^2^ = 0.33) through estimating genetic parameters, suggesting that HVP was significantly affected by genetic factors and was not a simple Mendelian trait. As shown in Table 1, HVP had significantly positive genetic correlations with growth and carcass traits in chickens (*P* < 0.05), such as body weight at day 91 (r_g_ = 0.27), carcass weight (r_g_ = 0.24), net weight (r_g_ = 0.27), breast muscle weight (r_g_ = 0.17), and leg muscle weight (r_g_ = 0.34). Moderately negative genetic correlations were identified between HVP and immune traits, especially the antibody response to Newcastle disease virus (r_g_ = −0.42). These results indicated that the immune capacity of chickens with HVP could be inferior to that of normal non-pigmented chickens, but the growth capacity of chickens with HVP might be greater.

**Table 1:** **Genetic correlations** (**r**_**g**_) **between the hyperpigmentation of visceral peritoneum and growth**, **carcass**, **and immune traits in chickens**

**Traits**^**a**^	**No.**^**b**^	**Means**^**c**^	**r**_**g**_	***P***-**value**
**BW91**, **g**	511	2,100 ± 16.5	0.27 ± 0.14	2.43 × 10^-2^
**CW**, **g**	511	1,847 ± 14.5	0.24 ± 0.14	4.83 × 10^-2^
**NW**, **g**	511	1,401 ± 11.7	0.27 ± 0.13	1.86 × 10^-2^
**DW**, **g**	506	1,674 ± 13.7	0.21 ± 0.16	9.47 × 10^-2^
**BMW**, **g**	511	122 ± 1.09	0.17 ± 0.10	3.84 × 10^-2^
**LMW**, **g**	511	168 ± 1.95	0.34 ± 0.10	4.50 × 10^-4^
**AFW**, **g**	510	82.6 ± 1.58	0.07 ± 0.12	2.72 × 10^-1^
**SIL**, **cm**	510	129 ± 0.59	−0.06 ± 0.10	2.66 × 10^-1^
**Ab**-**NDV**	511	3.63 ± 0.07	−0.42 ± 0.14	1.35 × 10^-3^
**Ab**-**AIV**	511	1.31 ± 0.05	−0.26 ± 0.16	5.21 × 10^-2^
**HC**	508	13.4 ± 0.38	−0.25 ± 0.18	8.24 × 10^-2^

^a^ BW91 = body weight at day 91, CW = carcass weight, NW = net weight, DW = dress weight, BMW = breast muscle weight, LMW = leg muscle weight, AFW = abdomen fat weight, SIL = small intestine length, Ab-NDV = antibody response to Newcastle disease virus, Ab-AIV = antibody response to avian influenza virus, HC = heterophil count, representing heterophil to lymphocyte ratio.

^b^ Number of samples for estimating genetic correlations.

^c^ Mean ± standard error.

### GWAS detection of SNPs associated with HVP

**Figure 2:**
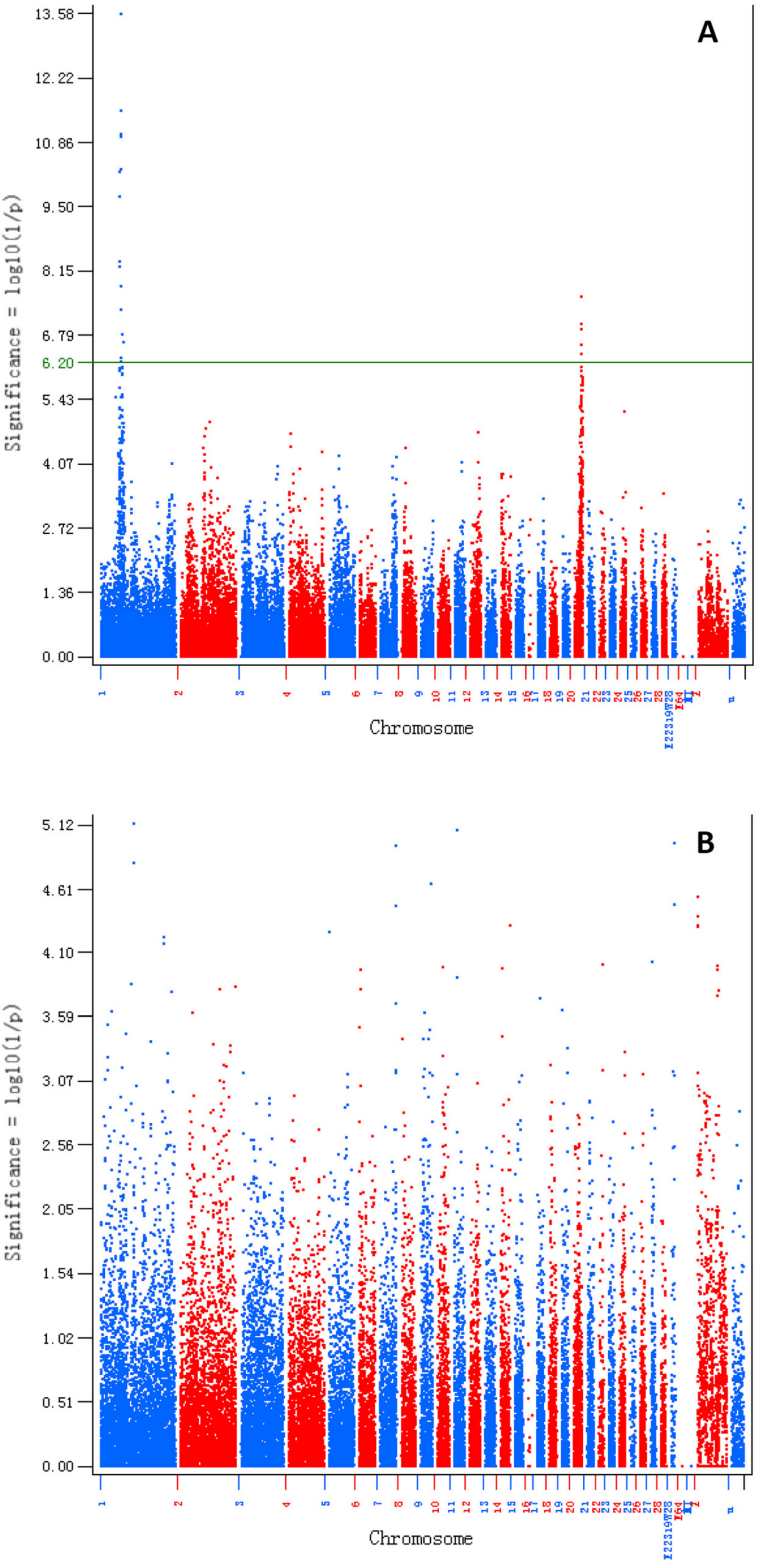
**Manhattan plot of the genome**-**wide association study for the hyperpigmentation of visceral peritoneum** (**HVP**) **in chickens.** The green line indicates the threshold *P* value of the 5% Bonferroni genome-wide significance (*P* = 6.28 × 10^-7^). **A**. Additive effects of GWAS for HVP. **B**. Dominance effects of GWAS for HVP.

A GWAS was used to dissect the genetic factors associated with HVP. SNP additive effect analysis identified several regions that were significantly (*P* < 6.28 × 10^-7^) associated with HVP on both GGA1 and GGA20 (Figure 2A). As shown in Table 2, 20 SNPs with additive effects with genome-wide significance were detected for HVP (*P* < 6.28 × 10^-7^). Thirteen SNPs with additive effects were located in the 50.5–54.0 Mb region of GGA1. In addition to this GGA1 region, seven SNPs with functional effects reached genome-wide significance, including two SNPs in the 58.5–60.5 Mb region of GGA1, and five SNPs in the 10.5–12.0 Mb region of GGA20. The extent of linkage disequilibrium (LD) in both GGA1 and GGA20 was about 2 Mb (Additional file 1: Figure S1), which indicated that the three significant regions represented three independent QTLs for HVP. The risk alleles of the QTLs in the 50.5–54.0 Mb region of GGA1 and in the 10.5–12.0 Mb region of GGA20 were from the parent broiler sire line and Huiyang Beard chicken line, respectively. The additive effect in the 50.5–54.0 Mb region of GGA1 was the greatest out of all the regions identified for HVP. The most significantly associated SNP (rs14822943) explained 13% of phenotypic variance for HVP. Because the heritability of HVP reached 0.33, the additive effect of this SNP covered 39% of additive genetic variance for HVP. Together with the most significant SNP in each strongly significant chromosomal region, the three most significant SNPs accounted for >50% of the additive genetic variance for HVP. Unlike the additive effect, no SNP dominance effect reached genome-wide significance (*P* < 6.28 × 10^-7^) for HVP (Figure 2B). This result indicated that the genetic factors affecting HVP had much stronger additive effects than dominance effects, and that at least three major genes could influence HVP.

**Table 2:** **SNPs with statistical significance in the genome**-**wide association study for hyperpigmentation of visceral peritoneum** (**HVP**)

**SNP**	**GGA**^**a**^	**Position (bp)**	**Nearest gene**^**b**^	**Alleles**	**RA**^**c**^	**FAW**	***P***-**value**	**AE**	**r**^**2**^
**rs14822943**	1	52,576,468	0.5 Kb U *MAP3K7IP1*	C/T	T	0.21	2.65 × 10^-14^	0.05	0.13
**GGaluGA017356**	1	52,358,970	*CACNA1I*	T/C	T	0.24	2.95 × 10^-12^	0.06	0.10
**GGaluGA017598**	1	52,940,122	*PLA2G6*	T/C	C	0.43	9.03 × 10^-12^	0.08	0.09
**GGaluGA018011**	1	53,833,467	70.8 Kb U *MYH9*	G/A	G	0.24	1.03 × 10^-11^	0.05	0.10
**rs13865536**	1	52,554,283	13.3 Kb D *MAP3K7IP1*	C/T	C	0.24	5.07 × 10^-11^	0.05	0.10
**GGaluGA017030**	1	51,619,588	0.8 Kb U *RANGAP1*	G/A	A	0.49	5.80 × 10^-11^	0.08	0.08
**rs13652125**	1	50,923,554	*TTC26*	A/G	A	0.15	1.97 × 10^-10^	0.04	0.06
**GGaluGA016761**	1	51,087,457	*SERHL2*	T/C	C	0.48	4.62 × 10^-9^	0.07	0.06
**GGaluGA016965**	1	51,488,752	2.5 Kb D *ACO2*	T/C	C	0.25	5.75 × 10^-9^	0.05	0.07
**GGaluGA017484**	1	52,642,092	*PDGFB*	G/A	G	0.26	1.52 × 10^-8^	0.05	0.09
**GGaluGA017730**	1	53,094,788	5.6 Kb U *TRIOBP*	T/C	T	0.64	4.74 × 10^-8^	0.11	0.09
**GGaluGA019336**	1	58,058,352	55.3 Kb U *HIPK2*	G/A	G	0.70	1.55 × 10^-7^	0.10	0.04
**rs13873173**	1	60,462,801	*SLC13A4*	G/T	T	0.75	2.37 × 10^-7^	0.10	0.05
**rs13865344**	1	52,399,455	*CACNA1I*	T/C	T	0.27	4.85 × 10^-7^	0.04	0.03
**rs13865892**	1	53,177,756	*LGALS2*	G/A	G	0.61	5.79 × 10^-7^	0.08	0.06
**rs16174305**	20	11,707,312	30.0 Kb D *BMP7*	C/T	C	0.42	2.44 × 10^-8^	0.06	0.05
**GGaluGA180727**	20	11,075,280	36.9 Kb U *STX16*	G/A	A	0.36	9.25 × 10^-8^	0.06	0.05
**rs14278900**	20	10,911,015	*GNAS*	G/A	A	0.37	1.24 × 10^-7^	0.05	0.05
**GGaluGA180980**	20	11,591,655	3.5 Kb U *RBM38*	G/A	A	0.44	2.59 × 10^-7^	0.06	0.03
**GGaluGA181122**	20	11,874,967	27.0 Kb D *TFAP2C*	T/C	C	0.48	3.98 × 10^-7^	0.06	0.05

^a^ GGA = chicken (*Gallus gallus*) chromosome;

^b^ Gene information from NCBI, 06 July 2011;

^c^ RA = risk allele related to HVP; FAW = frequency of the allele related to HVP in F_2_ population; AE = additive effect of SNP markers; r^2^ = contribution of each SNP to HVP.

### Positional candidate genes for HVP

As shown in the Table 2, there were 18 genes in close proximity to the 20 significant genome-wide SNP markers. The most significant effect was observed in the promoter region (about 0.5 Kb upstream) of TGF-β activated kinase 1/MAP3K7 binding protein 1 (*MAP3K7IP1*), located in the 50.5–54.0 Mb region of GGA1. Another SNP, located 13.3 Kb downstream of *MAP3K7IP1*, also had a genome-wide significant additive effect for HVP. Because HVP was associated with immune traits (Table 1), and because *MAP3K7IP1* is involved in some pathways associated with energy metabolism and immunity, such as the MAPK signaling pathway and the Toll-like receptor signaling pathway, *MAP3K7IP1* was identified as a potential positional candidate gene for HVP. The most significant effect of the other QTLs linked to HVP on GGA1 was at 55.3 kb upstream of the homeodomain interacting protein kinase 2 (*HIPK2*) locus. *HIPK2* participates in cell development, growth, and apoptosis, such as in the Wnt pathway, and was associated with HVP and chicken growth (Table 1), so was therefore also identified as a candidate gene for HVP. The most significant SNP on GGA20 was located nearest to bone morphogenetic protein 7 (*BMP7*), which can effect melanocyte growth and melanoma cell metastasis, and therefore *BMP7* was also chosen as one of the most important positional candidate genes for HVP. In addition, according to information from the Kyoto Encyclopedia of Genes and Genomes (KEGG), Gene Map Annotator and Pathway Profiler (GenMAPP), and Biocarta databases, *MAP3K7IP1*, *HIPK2*, and *BMP7* can all regulate mitogen-activated protein kinase kinase kinase 7 (*MAP3K7*) expression (Additional file 2: Figure S2), suggesting that each of these genes might affect HVP by mediating *MAP3K7* expression.

### Expression of chicken *MAP3K7IP1*, *HIPK2*, *BMP7*, and *MAP3K7*

**Figure 3:**
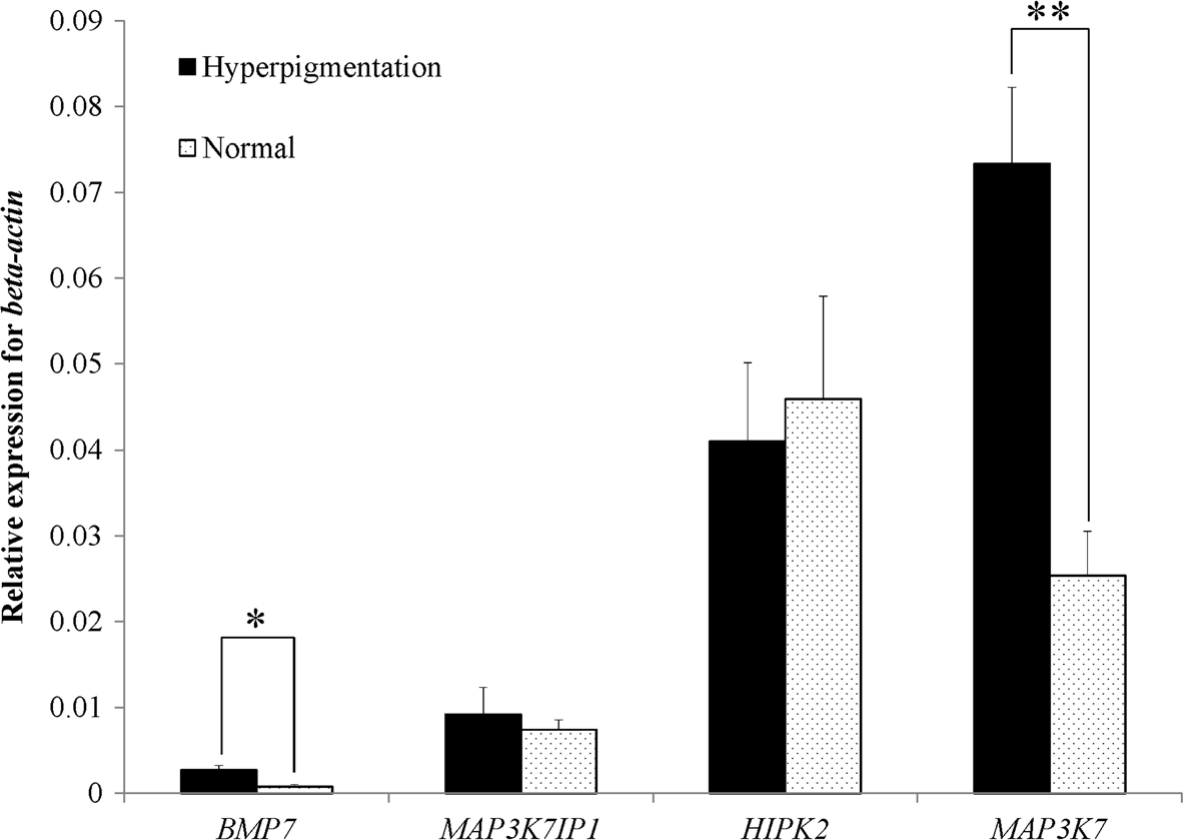
**Expression of chicken *MAP3K7IP1***, ***HIPK2***, ***BMP7***, **and *MAP3K7*.** * and ** indicate significant differences at *P* < 0.05 and *P* < 0.01, respectively, in gene expression between the visceral peritoneum of the normal Huiyang Beard chickens and Huiyang Beard chickens with hyperpigmentation of visceral peritoneum.

As shown in Figure 3, *MAP3K7IP1*, *HIPK2*, *BMP7*, and *MAP3K7* were all expressed in the visceral peritoneum tissue of normal Huiyang Beard chickens as well as in chicken with HVP. *BMP7* mRNA expression was the lowest, only slightly greater than zero in the normal Huiyang Beard chickens, while *MAP3K7* mRNA expression was the highest. The mRNA expression of both *BMP7* and *MAP3K7* in the visceral peritoneum tissue of Huiyang Beard chickens with HVP was significantly higher than that in normal Huiyang Beard chickens (*P* < 0.05). However, the mRNA expression of *MAP3K7IP1* and *HIPK2* was not significantly different between the visceral peritoneum tissue of normal Huiyang Beard chickens and those with HVP (*P* > 0.05).

## Discussion

Interestingly, chickens with HVP showed no reduction in body weight, and were actually associated with improved production efficiency. This finding deviated from our hypothesis that HVP was likely associated with disease phenotypes, which could decrease chicken growth. However, HVP-affected birds still had greater health risks because of the observed negative genetic correlations with immune traits (Table 1). The birds with HVP had decreased antibody responses, indicating that they may be more likely to suffer from pathogen infections. In addition, the birds with HVP had larger heterophil counts, resulting in higher heterophil to lymphocyte ratios (H/L). High H/L values in chickens are associated with decreased tolerance of environmental stress [25]. Taken together, our results indicated that birds with HVP should grow faster in a favorable environment, but stressful environmental conditions would more adversely affect the development and growth of birds with HVP compared with those of normal birds. We therefore inferred that the increased emergence of birds with HVP indirectly results from the selection of birds with higher production efficiency in the modern broiler industry.

During our investigation of HVP in chickens, we did not observe this HVP phenomenon in fast-growing birds with white feathers, such as Ross 308 broilers; HVP appeared to be limited to colored chicken breeds, such as Huiyang Beard chickens (unpublished data). The lack of the HVP phenotype in Ross 308 broilers may result from interference of the white dominance or recessive locus with the pigmentation in the abdominal septa of areolar connective tissues. A white recessive locus with a retroviral insertion in the tyrosinase (*TYR*) gene changes the expression of *TYR* to interrupt melanin biosynthesis [11,26,27], while a white dominance locus with a mutation in the premelanosome protein gene alters melanosome shape to influence pigmentation [10,28]. In addition, birds with runting and stunting syndrome (RSS) usually also have the HVP phenotype in Chinese farms (personal communication). One of reasons behind RSS may be the fact that birds with HVP have less resistance to pathogens and environmental stress, such as cold temperatures. Therefore, HVP may be one of the traits that mirror non-balance allocation of energy between production and immunity during chicken growth. However, this hypothesis required further experimental validation.

A GWAS was implemented for HVP in this study to attempt to validate the above hypotheses. GWAS are useful for exploring the genetic basis of some special appearance traits, such as pigmentation [29,30]. This was the first study aimed at uncovering the genetic basis of pigmentation of connective tissues in chickens based on a high-density SNP chip panel. We hoped that the findings would increase the genetic knowledge of HVP, and allow us to validate potential HVP candidate genes.

The GWAS identified 20 SNP markers that were significantly (*P* < 6.28 × 10^-7^) associated with HVP (Table 2). Based on the extent of LD on GGA1 (Additional file 1: Figure S1), these SNP markers were determined to belong to three different QTLs. The detection of more than one QTL indicates that the causal genes or mutations in these QTLs can affect the same pathway or gene to generate the same phenotype. The *MAP3K7IP1*, *HIPK2*, and *BMP7* genes were the closest loci to the most significant SNP marker in each of the three QTL regions. *MAP3K7IP1*, *HIPK2*, and *BMP7* genes are not traditional pigmentation genes, which are generally considered to include *MC1R*, *TYR*, tyrosinase-related protein 1, microphthalmia-associated transcription factor (*MITF*), agouti signaling protein, SRY (sex determining region Y)-box 10 (*SOX10*), myosin VA (heavy chain 12, *myoxin*), solute carrier family 45, and member 2 [8,31,32]. Therefore, we hypothesized that HVP does not directly result from mutations in the traditional pigmentation genes, but originates from the upstream genes that can indirectly change pigmentation pathways. GWAS of human pigmentation traits have produced some similar results [33]. Previous studies also suggested that *MAP3K7IP1*, *HIPK2*, and *BMP7* could influence some pigmentation pathways. For example, Liang et al. found that down-regulation of *HIPK2* expression suppressed the expression of *MITF*, resulting in melanocyte differentiation suppression by increasing C-terminal binding protein 2 levels [34]. BMP7 could inhibit normal melanocyte growth and tumor growth of human uveal melanomas [35,36], and could inhibit metastasis by inducing mesenchymal-to-epithelial transition in melanoma cells [37]. However, evidence indicates that *BMP7* is upregulated in the development of melanoma [38,39]. This study also found that upregulation of *BMP7* was associated with HVP in Huiyang Beard chickens (Figure 3). In addition, BMP7 could affect pheomelanin generation by interacting with proopiomelanocortin in brown adipocyte differentiation and thermogenesis [32,40].

More interestingly, the *MAP3K7* gene was found to be a node linking the *MAP3K7IP1*, *HIPK2*, and *BMP7* genes according to the pathway maps involving these genes in the KEGG, GenMAPP, and BioCarta databases (Additional file 2: Figure S2). MAP3K7 can also interact with many genes affecting melanocyte development [31,32], such as MITF [41], KIT ligand, B-cell leukemia/lymphoma 2 [42,43], lymphoid enhancer binding factor 1 [44], and epidermal growth factor receptor [45,46]. MAP3K7IP1 is one of the MAP3K7 binding proteins. The MAP3K7IP1 protein interacts with and thus activates MAP3K7 kinase, and may also function as a mediator between TGF-β receptors and MAP3K7 [47-49], suggesting that MAP3K7IP1 can influence the function of the downstream genes of the pathways involving in MAP3K7. Besides the interaction of MAP3K7IP1 and MAP3K7, BMP7 also contacts MAP3K7 in some pathways. Yamaguchi et al. reported that BMP7 activated MAPK signaling through MAP3K7 [50]. Blank et al. verified that BMP7 activated the JNK signaling pathway, and MAP3K7 was required for BMP7-mediated JNK activation [51]. In addition, BMP7 could activate MAP3K7 and enhance Wnt-dependent transcription [52]. Expression of *BMP7* was indeed consistent with that of *MAP3K7* in this study (Figure 3). It is possible that a specific mutation upregulates expression of BMP7 to result in MAP3K7 upregulation in Huiyang Beard chickens with HVP. Additionally, the MAP3K7-HIPK2 pathway can inhibit c-Myb activity upon Wnt-1 stimulation, affecting the immune response, because c-Myb plays an essential role in the proliferation of immature hematopoietic cells and early T-cell development [53-55]. This is consistent with the fact that this study identified a strong genetic relationship between HVP and the antibody response to Newcastle disease virus (r_g_ = −0.42, Table 1). Importantly, MAP3K7 participates in several pathways related to the immune response, such as the B cell receptor signaling pathway, the toll-like receptor signaling pathway and the IL-6 signaling pathway (http://www.wikipathways.org/index.php/WikiPathways). These findings indicate that *MAP3K7IP1*, *HIPK2*, and *BMP7* could be candidate genes for HVP, and might affect the development of HVP by regulating the expression of the *MAP3K7* gene. Further studies are needed to validate this hypothesis.

## Conclusions

HVP was found to be a quantitative trait with moderate heritability. Three independent QTLs for HVP were detected by GWAS on GGA1 and GGA20, and the *BMP7* gene was identified as a likely candidate gene for HVP.

## Methods

### Ethics statement

This study was approved by the Animal Care Committee of the Institute of Animal Science, Guangdong Academy of Agricultural Sciences (Guangzhou, People’s Republic of China), with approval number GAAS-IAS-2009-73. Animals involved in this study were humanely sacrificed as necessary to ameliorate their suffering.

### Animals and data collection

A total of 585 commercial chickens were used in this study, consisting of three generations (23 P, 51 F_1_, and 511 F_2_ individuals) with an accurate pedigree. All birds were immunized with a commercial avian influenza-inactivated H9 strain vaccine at day 40, and a commercial Newcastle disease virus live LaSota strain vaccine at day 50. At day 91, 511 F_2_ individuals from six hatches were slaughtered. At this time point, vein blood was collected and a portion transferred into centrifuge tubes containing ethylenediaminetetraacetic acid disodium salt solution, and then stored at −80°C. The remainder was used to prepare serum for measuring antibody responses (S/P values) to Newcastle disease virus and avian influenza virus by enzyme linked immunosorbent assay. At day 91, body weight, carcass weight, net weight, dress weight, breast muscle weight, leg muscle weight, and abdomen fat weight were measured, as was small intestine length. Heterophil count, representing H/L, was measured following the method of Vleck et al. [56]. A higher heterophil count is consistent with a higher H/L value. Because HVP was thought to be a quantitative trait, HVP was classified into three levels, absent, mild, and severe hyperpigmentation, represented by 0, 1, and 2 (Figure 1), respectively, to control for false positives. The absent, mild, and severe hyperpigmentation groups had 352, 132, and 27 individuals, respectively.

### SNP genotyping and selection

Genomic DNA extraction from venous blood was performed using the phenol/chloroform method. The quality and concentration of genomic DNA from 511 F_2_ individuals fulfilled the requirements for the Illumina Infinium SNP genotyping platform. Genotyping using the Illumina 60K Chicken SNP Beadchip [57] was carried out at the Illumina-certified service provider, DNA LandMarks, Saint-Jean-sur-Richelieu, Canada. Quality control was assessed in GenomeStudio v2008.1 [58]. Six samples were excluded as more than 5% of their SNPs had missing genotypes. The final SNP set included 39,833 SNPs for this GWAS under the following SNP selection criteria: low call frequency (>95%), low heterozygosity cluster intensity and separation value (>0.4), and low minor allele frequency (>0.1). Information on the SNP markers on each chicken chromosome is summarized in Additional file 3: Table S1.

### Gene expression

The visceral peritoneum tissue from eight normal and eight HVP-positive, 21-day-old Huiyang Beard chickens (a Chinese native chicken breed from Guangdong province) was collected and transferred into RNA*later* solution (Life Technologies, Carlsbad, CA, USA) and stored at −80°C. Total RNA was isolated by grinding the tissues to powder under liquid nitrogen and extracting with RNA TRIzol reagent (Life Technologies, Rockville, MD, USA). The RNA was reverse transcribed into cDNA with an M-MLV RTase cDNA Synthesis Kit (Takara Biotechnology Co., Dalian, China). Quantitative real-time polymerase chain reaction (qRT–PCR) analysis was performed to test the expression of *MAP3K7IP1*, *HIPK2*, *BMP7*, and *MAP3K7* in the visceral peritoneum tissue of the birds. The primers used for qRT-PCR were designed using Primer Express 2.0 software (Applied Biosystems, Foster City, USA) and their sequences are shown in Table 3. qRT-PCR reactions used SYBR Green Real Time PCR Master Mix (Toyobo Co., Osaka, Japan) according to the manufacturer’s instructions, and contained a passive reference dye, Rox, to correct for well-to-well variation. Reactions were run on a LightCycler 480 Real-Time PCR System (Roche Applied Science, Indianapolis, IN, USA) with the following parameters: 3 min at 95°C, followed by 40 cycles of 30 s at 95°C, 30 s at 60°C, and 34 s at 72°C. The relative mRNA expression of the target genes was measured as the number of cycles of PCR required for exceeding threshold fluorescence, and was normalized against that of *β*-*actin*, according to the quantitation procedures recommended by Roche Applied Science.

**Table 3:** **Sequences of primers used for qRT**-**PCR**

**Genes**	**Forward primers** (**5**′–**3**′)	**Reverse primers** (**5**′–**3**′)
*BMP7*	GAGAACAGCAGCAGCGACC	CAAAATAGAGCACTGAGATGGC
*MAP3K7IP1*	CCCCACCCTCACTAACCAA	TCCCTCCTCAGTCTTTTCTCAC
*HIPK2*	CATCCTCGGTTTACCATTTTG	CGGTGAGTCTGTATCCCTGTT
*MAP3K7*	CCAGGAAACGGACAGCAG	CTTTGGAGTTCGGGCATG
*β*-*actin*	CCCCAAAGCCAACAGAGAGA	GGTGGTGAAGCTGTAGCCTCTC

### Statistical analysis

Variance and covariance components were estimated using the average information restricted maximum likelihood algorithm [59] implemented by the DMU package [60]. The variance component estimated model was:

y=Xb+Za+e

where *y* was the vector of observations of HVP, e.g. body weight at day 91(a total of nine phenotypes, Table 1); *b* was the vector of fixed effects, including sex (two levels) and hatch (six levels); *a* was the vector of animal additive genetic effects; *e* was the vector of random residuals; and *X* and *Z* were corresponding incidence matrices.

Statistical tests of SNP-phenotype association were implemented using the generalized least square version of the epiSNP computer package, which considered sib correlation within each family [61,62]. The statistic model was

Y=μ+S+H+f+SNP+e,

where *Y* was the phenotypic value of HVP, *μ* was the common mean of HVP, *S* was the fixed gender effect, *H* was the fixed hatch effect, *f* was the random family effect, *SNP* was the single-locus SNP genotypic effect, and *e* was the random residual. Additive and dominance effects were tested using linear contrasts of the single-locus SNP genotypic effect [62]. The threshold *P* value of the 5% Bonferroni genome-wide significance was 6.28 × 10^-7^ (0.05/39833/2), based on the total number of SNP markers and two SNP genotypic effects (additive and dominance effects) in GWAS. Manhattan plots were produced using SNPEVG version 2.1 [63] to demonstrate the overview of SNP effects.

To evaluate the extent of LD and identify potential regions of causal mutation for HVP, pairwise LD, measured by r^2^ values for the F_2_ population, was calculated for GGA1 and GGA20 using Haploview [64]. Pathway analysis was performed using KEGG (http://www.genome.jp/kegg/), GenMAPP (http://genmapp.org/), and BioCarta (http://genmapp.org/) databases.

Differential expression of *MAP3K7IP1*, *HIPK2*, *BMP7*, and *MAP3K7* in the visceral peritoneum tissue, between the normal birds and the birds with HVP, was determined using a *t*-test with SAS 8.0 software (SAS Institute, Cary, NC, USA).

## Competing interests

The authors have no competing interests to declare.

## Authors’ contributions

CL, HQ, XH, NL, and DS conceived and designed the experiments. CL, HQ, JW, YW, JM, CYL, and DS performed the experiments. CL and HQ analyzed the data. CL, JM, CY, and DS contributed the materials. CL, HQ, and DS wrote the paper. All authors read and approved the final manuscript.

## Supplementary Material

Additional file 1: Figure S1 Pattern of linkage disequilibrium (LD) on chicken (*Gallus gallus*) chromosomes. **A**. LD on Chromosome 1. **B**. LD on Chromosome 20. Click here for file

Additional file 2: Figure S2 Interaction of genes. Blue, green, and red figures indicate the number of pathways that the genes are involved in, based on KEGG, GenMAPP, and BioCarta, respectively. BMP7 interacts with MAP3K7 via the ALK pathway in cardiac myocytes according to the BioCarta database. HIPK2 interacts with MAP3K7 via an enzyme linked receptor protein signaling pathway and Wnt netPath 8 according to the GenMAPP database. MAP3K7IP1 interacts with MAP3K7 via the MAPK and Toll-like receptor signaling pathways according to the KEGG database, by the NF-κB signaling pathway, signal transduction through IL1R, the TGF-β signaling pathway, and the WNT signaling pathway according to the Biocarta database, and by receptor signaling protein activity, the MAPK signaling pathway, and TGF-β-receptor netPath 7 according to the GenMAPP database. Click here for file

Additional file 3: Table S1. Basic information on the 39,833 SNP markers on the chicken physical map. Click here for file
