# Supplementary figures and images for: Genetic parameters and genome-wide association study of hyperpigmentation of the visceral peritoneum in chickens
Source: BMC Genomics. 2013 May 16;14:334. doi: 10.1186/1471-2164-14-334 (PMC3663821; doi:10.1186/1471-2164-14-334)

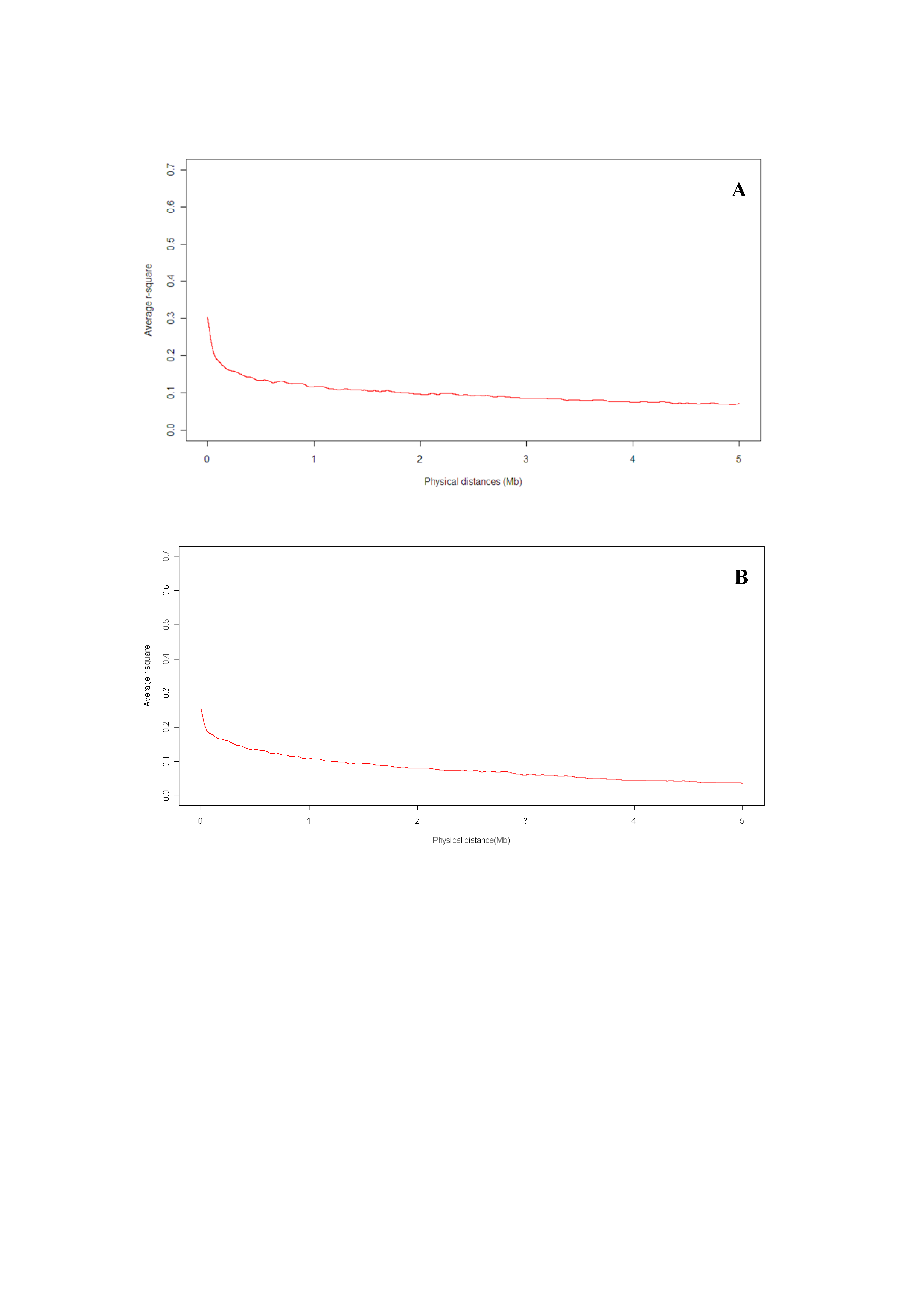

Supplement: Additional file 1: Figure S1 — Pattern of linkage disequilibrium (LD) on chicken (Gallus gallus) chromosomes. A. LD on Chromosome 1. B. LD on Chromosome 20. [file 1471-2164-14-334-S1.tiff]

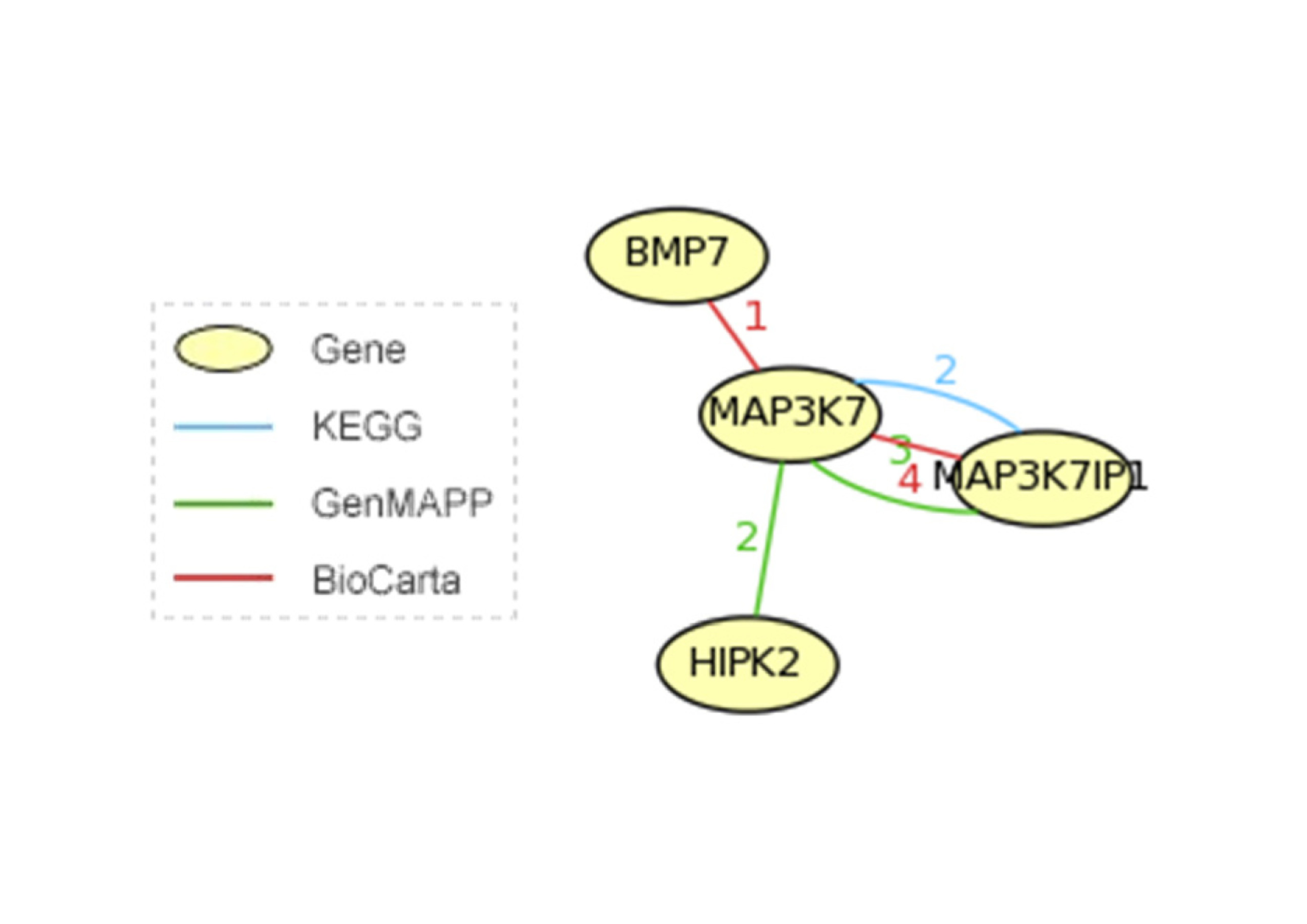

Supplement: Additional file 2: Figure S2 — Interaction of genes. Blue, green, and red figures indicate the number of pathways that the genes are involved in, based on KEGG, GenMAPP, and BioCarta, respectively. BMP7 interacts with MAP3K7 via the ALK pathway in cardiac myocytes according to the BioCarta database. HIPK2 interacts with MAP3K7 via an enzyme linked receptor protein signaling pathway and Wnt netPath 8 according to the GenMAPP database. MAP3K7IP1 interacts with MAP3K7 via the MAPK and Toll-like receptor signaling pathways according to the KEGG database, by the NF-κB signaling pathway, signal transduction through IL1R, the TGF-β signaling pathway, and the WNT signaling pathway according to the Biocarta database, and by receptor signaling protein activity, the MAPK signaling pathway, and TGF-β-receptor netPath 7 according to the GenMAPP database. [file 1471-2164-14-334-S2.tiff]
